# Reconstruction of noise-driven nonlinear networks from node outputs by using high-order correlations

**DOI:** 10.1038/srep44639

**Published:** 2017-03-21

**Authors:** Yang Chen, Zhaoyang Zhang, Tianyu Chen, Shihong Wang, Gang Hu

**Affiliations:** 1 School of Sciences, Beijing University of Posts and Telecommunications, Beijing, China; 2 Faculty of Science, Ningbo University, Ningbo, China; 3 Department of Physics, Beijing Normal University, Beijing, China

## Abstract

Many practical systems can be described by dynamic networks, for which modern technique can measure their outputs, and accumulate extremely rich data. Nevertheless, the network structures producing these data are often deeply hidden in the data. The problem of inferring network structures by analyzing the available data, turns to be of great significance. On one hand, networks are often driven by various unknown facts, such as noises. On the other hand, network structures of practical systems are commonly nonlinear, and different nonlinearities can provide rich dynamic features and meaningful functions of realistic networks. Although many works have considered each fact in studying network reconstructions, much less papers have been found to systematically treat both difficulties together. Here we propose to use high-order correlation computations (HOCC) to treat nonlinear dynamics; use two-time correlations to decorrelate effects of network dynamics and noise driving; and use suitable basis and correlator vectors to unifiedly infer all dynamic nonlinearities, topological interaction links and noise statistical structures. All the above theoretical frameworks are constructed in a closed form and numerical simulations fully verify the validity of theoretical predictions.

In recent decades, the topic of dynamical complex networks has attracted great attention in interdisciplinary fields due to its theoretical importance and practical significance^1^,^2^. Network dynamics is determined in great extent by network structures, mainly classified by dynamics of local nodes and interactions between network nodes. In many practical cases, we can measure outputs of network nodes while structures of networks are often deeply hidden in the measured data. Therefore, it turns to be crucial to develop effective methods to infer network structures from the available data of nodes. This is the so-called inverse problem of network reconstruction, which has become one of the most important topics in the data analysis of complex networks in wide crossing fields, particularly in biological and social sciences^3^,^4^,^5^,^6^,^7^.

Various methods have been proposed to treat network reconstruction problems^8^. There are several typical difficulties in practice. First, in most of realistic cases diverse facts, such as noises, are involved in the data production. These noises make the structure inference difficult because they are unknown on one hand, and essentially influence the data analysis on the other hand. Different statistical methods based on various correlation computations have been suggested to treat the noise problem^9^,^10^,^11^,^12^,^13^,^14^. Second, in almost all realistic network systems various nonlinearities play crucial roles in generating diverse characteristic features and significant functions. So far most of works in treating network inference have made approximations either neglecting noise influences^15^,^16^,^17^,^18^,^19^,^20^,^21^,^22^,^23^,^24^,^25^, or considering linear dynamics and interactions^9^,^10^,^11^,^12^,^13^,^14^. These methods fail when both noise effects and nonlinearities of network structures are crucial for the data production. There have been few works treating the two difficulties jointly^26^,^27^,^28^,^29^,^30^, among which the Bayesian approach was used to estimate noise levels and dynamic parameters. In particular, a method of improved Bayesian method has been suggested for reconstruction of stochastic nonlinear dynamical model by iterating a number of coupled and nonlinear matrix equations with unknown dynamical parameters and noise statistical quantities^28^,^29^,^30^. The algorithm is analytical, but complicated if networks are large and noises at different nodes are coupled and multiplicative.

In this presentation we consider the problem of reconstruction of noise-driven nonlinear dynamic networks. The key points in dealing with the difficulties are: We compute high-order correlations to treat possible nonlinear structures; We use two-time correlations to separate the reconstructions of dynamical networks and noise statistics to two independent steps, and finally the computation of inference of noise-driven nonlinear networks has been converted to simple linear and algebraic matrix equations. In the first part of the section Results and Discussion, the idea and the method how to use high-order correlation computation (HOCC) to infer nonlinear dynamic networks are explained, including inferring all the internal node dynamics, mutual interactions and statistical structures of multiplicative noises. In the next part we first apply the HOCC method to a simple three-node network, the well known Lorenz equation, driven by white additive and multiplicative noises. And then large noise-driven complex networks with diverse nonlinear local dynamics and complicated links between nodes are considered. The effectiveness of the HOCC algorithm are well justified for both model networks. In the third part networks with complicated nonlinear phase dynamics, nonlinear interactions and nonlinear multiplicative noises are investigated. The HOCC method also works well in this more complicated case. To the end some conclusion and perspective of practical applications of the method are given. In the section of Materials and Methods, some details on the calculation of statistics of multiplicative noises are specified. Moreover, the problems how to treat measurement noises and colored noises and how reconstruction errors of the HOCC method depend on data length, system size and choice of basis sets are also briefly discussed (see Supplementary Information).

## Results and Discussion

### Inferring nonlinear networks by using high-order and two-time correlations: theory

Let us consider a general noise-driven nonlinear network


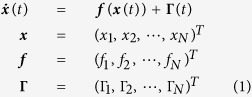


where T represents the operation of transposition. Dynamical noises Γ_*i*_(*t*), *i* = 1, 2, 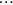, *N*, represent the impacts from microscopic world, and they are expected to have very short correlation time *τ*_*d*_ ≪ 1, much smaller than the characteristic time of deterministic dynamics assumed to be of order 1. Then noises are approximated as white ones,


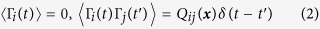


with *i, j* = 1, 2, 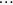, *N*. It is emphasized that multiplicative noises have been seldom considered so far in the study of network reconstruction, though this type of noises exist extensively in practical circumstances^31^,^32^,^33^,^34^. In Eq. (1) we have all measurable data in our hand, namely, we measure


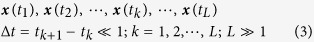


With Δ*t* ≪ 1 we can compute velocities of ***x*** in Eq. (1) and with 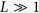 we have sufficiently large samples to perform statistical analysis. These conditions are not always available, but they do be available in many important practical experiments, or can be realized on purpose in case of need in many realistic measurements.

In Eq. (1) all functions *f*_*i*_, *i* = 1, 2, 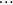, *N*, are unknown. The noise statistic matrix 
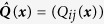
 is also unknown. Only the output variables (3) are available for analysis. The task is to specify dynamic functions *f*_*i*_, and noise statistics *Q*_*ij*_, *i, j* = 1, 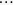, *N*.

First we assume *f*_*i*_s can be generally expanded by a certain basis set^16^,^24^,^30^,^35^


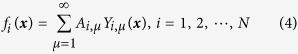


where all constant coefficients *A*_*i,μ*_, *μ* = 1, 2, 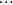, *M*_*i*_, 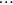, ∞ are unknown, while all functions *Y*_*i,μ*_(***x***) called as bases are known. For treating nonlinearities in ***f**(**x***), the chosen basis set should be complete for expanding field ***f**(**x***). In Eq. (1) we give a freedom to use different basis sets for expanding different field ***f**(**x***). It seems that the expansions of Eq. (4) should include infinite terms for arbitrary functions ***f**(**x***). One has to truncate the expansion to finite terms. There is a systematical and self-consistent method, described in the following part to make such truncation. At present, we just assume a truncation at *M*_*i*_ for *f*_*i*_(***x***) expansion. Then Eq. (4) can be simplified as


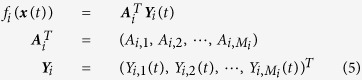


Without noise Γ_*i*_(*t*), all the unknown coefficients can be solved by algebraic equations if sufficient data are accumulated^16^,^21^. With strong noises the inference computations are much more difficult. Here for network reconstruction we use a method to compute two-time correlations to filter noise effect^11^,^14^, together with using high-order correlations of the chosen basis set to reconstruct interactions and nonlinearities of the networks and multiplicative noises.

For arbitrary node *i* in the network, multiplying Eq. (1) from the right side by a functional vector 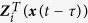



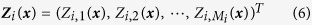


and computing all related correlations, we obtain a linear matrix algebraic equation


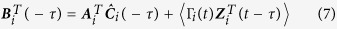


with


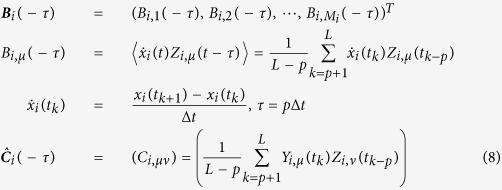


***Z***_1_(***x**(t*)), ***Z***_2_(***x**(t*)), 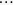, ***Z***_*N*_(***x**(t*)) are called correlators. They can be arbitrarily chosen for computing correlation matrix 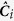 under the condition that their entries must not be linearly dependent on each other so that matrix 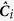 has full rank, and is invertible. In Eq. (8) we should have 0 ≈ *τ*_*d*_ ≪ *τ* ≪ 1 with *τ* being larger than the correlation time of dynamical noises *τ*_*d*_, and much smaller than the characteristic times of deterministic network dynamics, previously assumed to be of order 1. Therefore, noises and correlators must be decorrelated


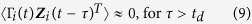


since the fast-varying noises of Eq. (2) have no correlation with any variable data of earlier times, disregarding any forms of multiplicative noises *Q*_*ij*_(***x***). Now with the noise-decorrelation of Eq. (9), Eq. (7) can be reduced to


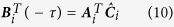


leading to


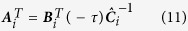


where ***B***_*i*_(−*τ*), 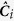 and ***A***_*i*_ are given in Eqs (8) and (5), respectively. We delete the notion (−*τ*) in 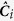 because *τ* ≪ 1 does not considerably change the values of *C*_*i,μν*_. All elements of vector ***B***_*i*_(−*τ*) and matrix 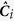 can be computed with known output variables ***x**(t*), and thus all the unknown linear and nonlinear coefficients in Eq. (1) can be inferred with a simple and direct linear matrix equation (11), though the noise matrix 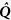 in Eq. (2) is unknown.

Statistical features of noises play crucial roles in the data production. Inferring noise statistics is also important for understanding the nature of data and for predicting the future data production in practical cases. It is desirable that noise matrix 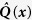 can be also easily reconstructed from the variable data by HOCC algorithm similar to the above


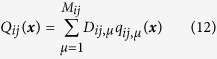



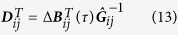


where


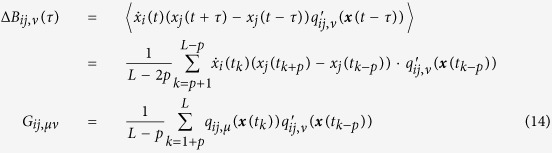


where *q*_*ij,μ*_(***x***) are known bases for expanding *Q*_*ij*_(***x***); *G*_*ij,μν*_ are matrix elements computable from correlations of basis *q*_*ij,μ*_ and corresponding correlator 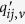; and *D*_*ij,μ*_ are unknown coefficients to be inferred. The detailed derivation of Eqs (13) and (14) is presented in Materials and Methods.

For numerical simulation we can arbitrarily choose correlator vectors and in the following computations we simply take,


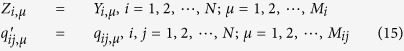


where *q*_*ij,μ*_(***x***), *μ* = 1, 2, 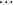, *M*_*ij*_ play role of vector bases for expanding multiplicative factor *Q*_*ij*_(***x***), and *M*_*ij*_ is the truncation in *Q*_*ij*_(***x***) expansion like *M*_*i*_ for *f*_*i*_(***x***).

Now four points of the present method should be emphasized. (i) High-order correlations are used to reconstruct nonlinear structure of networks without pursuing any linearization approximation; (ii) Two-time correlations have been proposed to decorrelate noise effects, and the time difference *τ* can be adjusted to suit different noise conditions; (iii) Basis set ***Y***_*i*_, ***q***_*ij*_ and correlator set 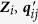 can be freely chosen to construct correlation matrices, suitable for different natures of practical networks. (iv) Multiplicative noises are taken into account in the study of network reconstruction problems, and multiplicative factors can be inferred together with the nonlinear fields and interaction structures. Unlike the multiple nonlinear algebraic equations of unknown matrices in ref. 30, where all network nodes *f*_*i*_, *i* = 1, 2, 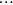, *N* and matrix elements *Q*_*ij*_, *i, j* = 1, 2, 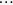, *N* are coupled and should be considered as a whole, here for the same noise-driven systems, we have derived two independent linear matrix equations Eqs (11) and (13), separately for each node *f*_*i*_ and each noise correlation element *Q*_*ij*_(***x***). Each of them can be explicitly solved node by node and element by element with available variable data. This advantage can extremely simplify computations when networks are large and noises are coupled and multiplicative.

### Inference of noise-driven networks by using power expansions

We first consider a three-node nonlinear network, the Lorenz system, one of the most famous models in chaos study^36^,


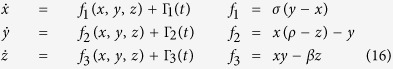


**Figure 1:**
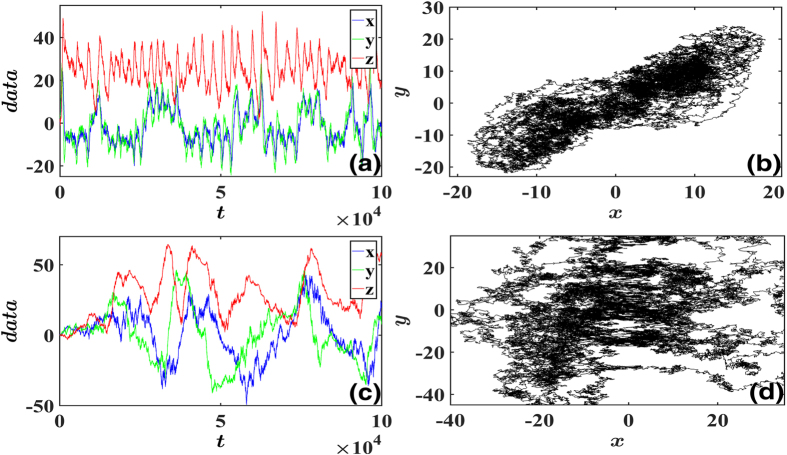
Time sequences and trajectories of noise-driven Lorentz system. Parameters are taken as *σ* = 10, *ρ* = 28, *β* = 8/3. (**a**,**b**) Additive noises *Q*_*ij*_ = 50*δ*_*ij*_ are applied. (**c**,**d**) Multiplicative noises *a*_1_ = 20, *a*_2_ = 50, *b*_1_ = 25, *b*_2_ = 60, *c*_1_ = 15, *c*_2_ = 30 in Eq. (17) are applied. Data are chaotic and strongly random. The trajectory in (**d**), from which one cannot see any trace of Lorentz dynamics, is considerably different from that of (**b**) for different noise factors, though Lorentz dynamic field is not changed in all (**a**–**d**).

Though network (16) looks very simple and low-dimensional, it serves as one of the most prevalent prototypes for data analysis. We consider noises


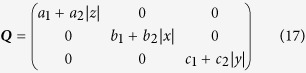


where we adopt absolute values |*x*|, |*y*|, |*z*| in *Q*_*ii*_ functions for *Q*_*ii*_ should be positive definite or zero from the definition of Eq. (2). In Eq. (16) both noise and nonlinearity essentially effect and noises with different multiplicative factors can produce considerably different data sets, as shown in Fig. 1 where trajectories for additive noises (Figs (a)(b)) and multiplicative noises (Figs (c)(d)) are presented. Now we apply the general algorithms Eqs (11) to this system. First we should choose bases for field expansion. Without particular information, power series can be naturally chosen as a candidate basis set. Then we assume the following bases with truncation *M*,


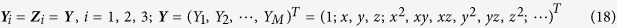


Here we introduce an idea of self-consistent checking of the truncation. At first we take 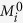 bases in Eq. (18), compute all elements of corresponding 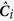, ***B***_*i*_ and obtain 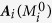. Then we further consider some more bases, i.e., 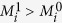 bases in Eq. (18), and obtain results of 
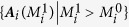
. 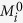 is concluded as a suitable truncation if the results satisfy two conditions. Condition (i): 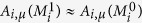 for all coefficients obtained with 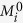 truncation; Condition (ii): 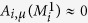 for all coefficients of bases not included by 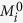 truncation. If any of the above two conditions is not satisfied we should go on to include more bases in (18), 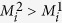, 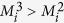 and so on, till the two conditions are satisfied at 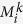 truncation. We then conclude 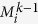 is the suitable truncation. Now we use the above method of truncations, successively from low orders to high orders of Eq. (18).

**Figure 2:**
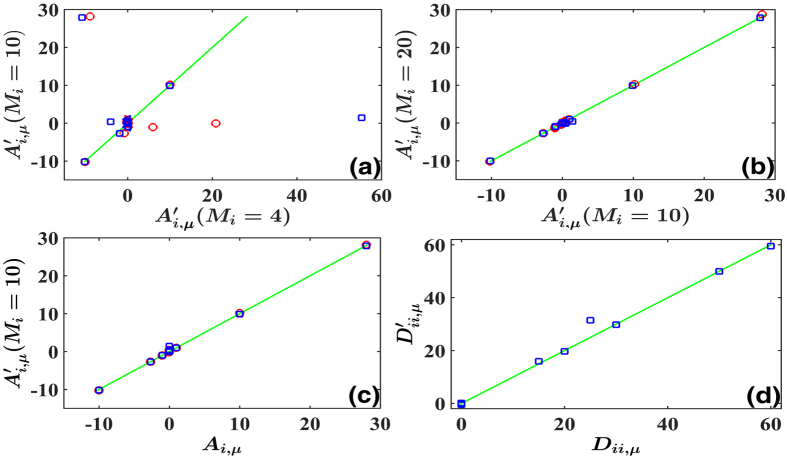
Demonstration of self-consistent checking of HOCC method, and its application in inference computations of Eq. (16). Red circles are for additive noises while blue squares for multiplicative ones. (**a**) Reconstruction results obtained by Eq. (11) with the truncated bases of the zero and the first orders of powers (*M*_*i*_ = 4) plotted against those with power bases truncated up to the second order *M*_*i*_ = 10. (**b**) The same as (**a**) with power bases truncated at the third order (*M*_*i*_ = 17) plotted against those truncated at the second order (*M*_*i*_ = 10). The truncation at *M*_*i*_ = 10 is self-consistently confirmed to be suitable. (**c**) Inference results obtained by Eq. (11) with *M*_*i*_ = 10 plotted against the actual coefficients. All plots are around the diagonal line, indicating correct reconstruction at *M*_*i*_ = 10. (**d**) *D*_*ii,μ*_ computed by Eq. (13) plotted against actual ones. Both sets coincide each other approximately.

A self-consistent justification on correct truncation for Eq. (16) is illuminated in Fig. 2. In Fig. 2(a), it is apparent that *M*_*i*_ = 4 (bases: 1, *x, y, z*) is not a proper truncation, since the reconstruction of *M*_*i*_ = 4 is considerably different from that of *M*_*i*_ = 10, where all bases of powers of the second order are taken into account. In Fig. 2(b) we compare the reconstruction results truncated by the second order (*M*_*i*_ = 10) with those of the third order (*M* = 20), and the above self-consistent checking conditions (i) and (ii) are both fulfilled. Therefore we can conclude it is enough to truncate the expansion at *M*_*i*_ = 10 with properly chosen bases in Eq. (18). In Fig. 1(c) we compare the reconstructed results for *M*_*i*_ = 10 with the actual *A*_*i,μ*_, and the nonlinear and interactive structures of Eq. (16) are satisfactorily recovered indeed.

In principle, the derivations from Eq. (4) to Eq. (11) and the computations of Fig. 2 can be conducted generally without knowing any trace of the network dynamics. The trials of truncations can be performed order by order from lower powers to high powers until successful self-consistent checking. However, the number of tested bases *K* increases very quickly by increasing the truncation order. For large networks *N* ≫ 1 we usually have *K* ≫ *N* ≫ 1, if we do not have any idea about the field ***f**(**x***) and the truncation has to be performed at large expansion order. For a given sample number *L* the reconstruction errors increases quickly as *L/K* decreases. Therefore, effectively reducing the number of tested bases *K* can make reconstruction less time consuming and more precise. There exist some methods to treat this problem. For instance, the compressive sensing method can exclude many zero coefficients from the inference computations and effectively reduce parameter number *K*^37^,^38^. This will not be discussed in the present paper.

For inferring noise statistics we construct expansion in the similar ways with bases Eq. (15)


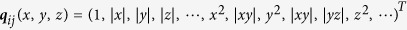


By applying Eqs (13) and (14) the coefficients of multiplicative factors of noises *D*_*ij,k*_ can be computed. The results are shown in Fig. 2(d). It is remarkable that with the randomly behaved data of Fig. 1 only we can correctly explore not only the nonlinear fields and interaction structures, but also the detailed multiplicative behaviors of noises.

**Figure 3:**
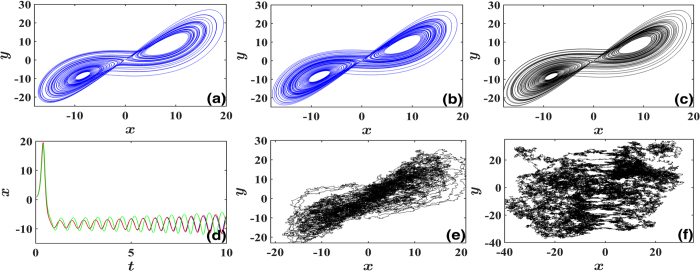
Comparisons of dynamics of reconstructed and original networks of noise-driven Lorentz system. (**a**–**c**) Comparison of the reproduced trajectories of the system reconstructed by our HOCC method ((**a**,**b**) for additive and multiplicative noises, respectively) with that of the original Lorentz system (**c**). Agreement is striking since only the strongly noisy data of Fig. 1 are used for dynamics reconstruction, and the data in Fig. 1(a,b) are so different from those in Fig. 1(c,d). (**d**) *x(t*) time sequences of (**a**–**c**) from the same position of *x* = 1, *y* = 0.1, *z* = 1. Blue, green and red lines correspond to time sequences of *x* variables in (**a**–**c**), respectively. The three trajectories run together for certain time interval, justifying the correct dynamics reconstruction for both additive and multiplicative noises. They diverge from each other, however, as time goes on due to the chaoticity of the dynamics. (**e**,**f**) Reproduced trajectories by noise-driven models inferred by HOCC with corresponding additive (**e**) and multiplicative (**f**) noises. The different characteristics of data in Fig. 1(b,d) are reproduced convincingly in (**e,f**) respectively.

We can also use the inference results to reconstruct the Lorentz network, simulate the predicted deterministic equations (***f*** only, without noise) and compare the trajectories with the original one with noise lifted (Fig. 3(a–d)). It is found that both reconstructed patterns match the original one well, though the two measured trajectories of Fig. 1(a–d) differ from each other considerably due to different multiplicative factors of noises. This coincidence justifies well the effectiveness of HOCC method. In Fig. 3(e,f) we produce variable data by simulating the reconstructed networks with derived noises with the inferred multiplicative factors. The features of the noisy data are reconstructed perfectly as well for both additive and multiplicative noises.

Next we infer a network with much higher dimension and more complicated nonlinearities


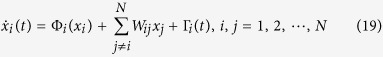


Equation (19) is very common in practical systems where local dynamic of each node is strongly nonlinear, and dynamic structures are diverse for different nodes. On the other hand interactions between nodes, the external facts for each node, are approximately linear. Expanding Φ_*i*_(*x*_*i*_) by power series to a power 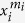



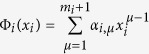


and referring to Eqs (1) and (4), we have


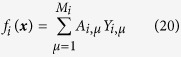


Then we can represent both unknown nonlinear structure and linear interaction topology unified as


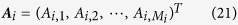


and define basis vector as


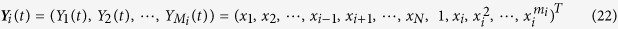


Choosing correlator vector ***Z***_***i***_ as ***Z***_***i***_ = ***Y***_***i***_, we can specify vector ***B***_*i*_(−*τ*) and matrix 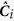 as


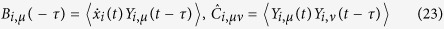


Inserting Eq. (23) into Eq. (11) we can solve all the unknown elements, including all the nonlinear and linear terms.

**Figure 4:**
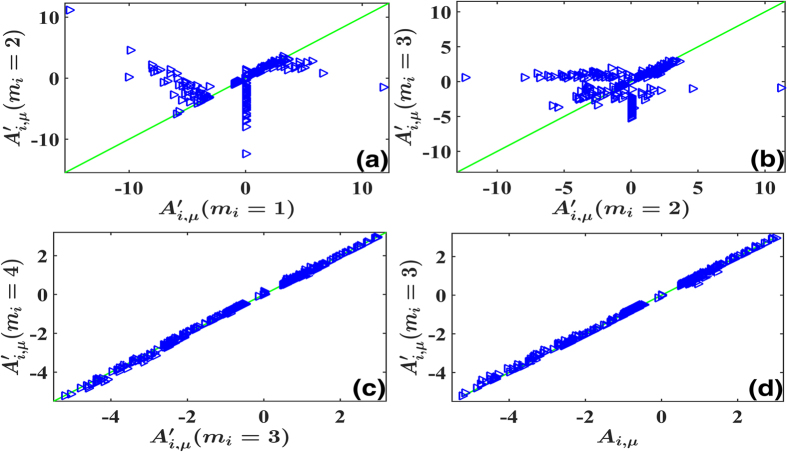
The same as Fig. 2 with network (19) of *N* = 50 considered. Parameters are taken with uniform distributions in the intervals *a*_*i*_ ∈ (−1, 1), *b*_*i*_ ∈ (2, 5) and *c*_*i*_ ∈ (0, 2) and additive noises *Q*_*ij*_ ∈ *δ*_*ij*_(0.5, 1). The interaction intensities are *W*_*ij*_ (*i, j* = 1, 2, 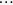, *N, i* ≠ *j*) ∈ (0.5, 1) with 10% probability for positive ones; ∈(−1, −0.5) also with 10% for negative ones; and *W*_*ij*_ = 0 otherwise. Power bases are organized from low-order to higher-order ones as described in Eq. (22). (**a**) Results of HOCC for *m*_*i*_ = 2 plotted vs. those for *m*_*i*_ = 1. Triangles are considerably away from the diagonal line, representing incorrect truncation at *m*_*i*_ = 1. (**b**) The same as (**a**) with results of *m*_*i*_ = 3 plotted with those of *m*_*i*_ = 2. The truncation at *m*_*i*_ = 2 is not suitable either. (**c**) Results for *m*_*i*_ = 4 plotted vs. those for *m*_*i*_ = 3. Both results coincide with each other fairly well, that self-consistently confirms the correctness of the HOCC method for sufficiently large *m*_*i*_, *m*_*i*_ ≥ 3. (**d**) The results of HOCC for *m*_*i*_ = 3 plotted vs. the actual values of *A*_*iμ*_. With suitable nonlinearity considered, all nonlinear and interacting structures are inferred correctly with certain fluctuations.

In Fig. 4 we investigate a particular case with local dynamics


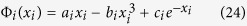


with *a*_*i*_, *b*_*i*_, *c*_*i*_ uniformly distributed as *a*_*i*_ ∈ (−1, 1), *b*_*i*_ ∈ (2, 5), *c*_*i*_ ∈ (0, 2). The network has mutual interactions (*i* ≠ *j*) positive *W*_*ij*_ ∈ (0.5, 1), *i, j* = 1, 2, 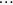, *N*, with 10% probability, negative ones  ∈ (−1, −0.5) also with 10%, and *W*_*ij*_ = 0 otherwise. Simple additive white noises are used for this model:


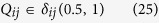


with uniform probability distribution. We consider different truncations *m*_*i*_’s. It is obvious that the reconstruction has large errors for too small *m*_*i*_ in Fig. 4(a,b) and they can be quickly improved by increasing *m*_*i*_, and saturate at sufficiently large *m*_*i*_ (Fig. 4(c,d), *m*_*i*_ = 3 is a fairly good approximation in our case). Unlike Eq. (16) where the power expansion of field ***f*** contains only powers lower than the third order and the truncation up to the second order is exact, the expansion of Eq. (24) has nonzero coefficients for infinitely large *m*_*i*_’s. And therefore, the satisfactory reconstruction results of Fig. 4(d) convincingly confirm that the approach of self-consistent truncation works stably also for nonzero while convergent expansions.

### Nonlinear network reconstruction by using different basis vectors

For inferring linear networks, the basis vectors can be simply chosen as output variables ***Y*** = (*x*_1_, *x*_2_, 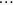, *x*_*N*_). For nonlinear networks, the ways to choose basis vectors become diverse. In Eqs (18) and (22) power expansions are used for representing nonlinear functions. Different types of basis vectors can be used, depending on the nature of data and property of nonlinear dynamics. In many practical cases the inverse computations of networks can be much more simplified by selecting suitable basis vectors. Let us consider a network of coupled Kuramoto model^25^,^26^,^28^,^29^,^39^ that has been extensively studied for describing oscillatory complex systems.


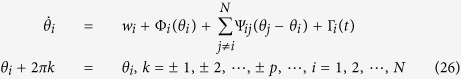


where *θ*_*i*_, *i* = 1, 2, 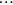, *N*, represent phase angles of oscillators, and all Φ_*i*_(*θ*_*i*_), Ψ_*ij*_(*ϕ*) are unknown nonlinear functions with topology


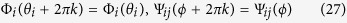


It is not convenient to approximate functions Eq. (27) by power expansions while we can conveniently do it by using Fourier basis vectors. Expanding Φ_*i*_, Ψ_*ij*_ as


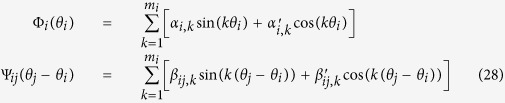


we can then define basis vector as


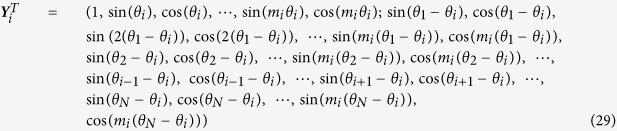


and the corresponding unknown coefficient vector as


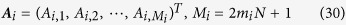


The correlator vector ***Z***_*i*_ can be simply defined as


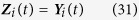


Inserting Eq. (29) for ***Y**(t*) and Eq. (31) for ***Z**(t* − *τ*) (0 < *τ* ≪ 1) into Eq. (11), we can specify all elements of vector ***B***_*i*_(−*τ*) and matrix 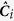, and explicitly infer all the nonlinear structures and interaction links of targeted vector ***A***_*i*_ in Eq. (30).

**Figure 5:**
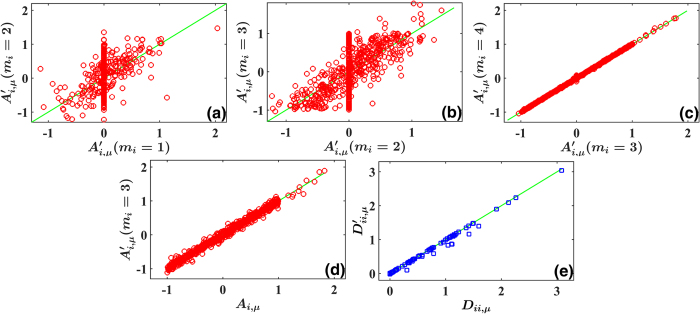
The same as Fig. 2 with network Eq. (26) reconstructed by using Fourier basis set. *N* = 10, and other parameters are uniformly distributed on *A*_*i,μ*_ ∈ (−1, 1) (for all nonzero coefficients in Equation(32), *μ* ≥ 2 and *m*_*i*_ ≤ 3), *A*_*i*,1_ ∈ (1, 2) and all the Fourier components of *m*_*i*_ ≥ 4 are zero. Multiplicative noises Eq. (33) are used with *a*_*ij*_, *b*_*ij,k*_ ∈ (0, 1). Truncations are made from low-order harmonics to higher-order ones. (**a**,**b**) The same as Fig. 2(a) with phase dynamics Eq. (26) considered, and the results of *m*_*i*_ = 2 plotted vs. those of *m*_*i*_ = 1 in (**a**); *m*_*i*_ = 3 vs. *m*_*i*_ = 2 in (**b**). Without high-order harmonics, many dots distribute away from the diagonal lines. (**c**) The results of *m*_*i*_ = 4 plotted vs. those of *m*_*i*_ = 3. All dots are distributed around the diagonal line, indicating self-consistently the suitable truncation at *m*_*i*_ = 3. (**d**) The same as Fig. 2(c) with Eq. (26) computed. All computed results at *m*_*i*_ = 3 coincide with those of the actual *A*_*i,μ*_ with certain fluctuations. (**e**) The same as Fig. 2(d) with noises (33) computed. The results of multiplicative noises 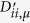 agree rather well with actual noise parameters *D*_*ii,μ*_’s.

We take a network of *N* = 10 as an example with


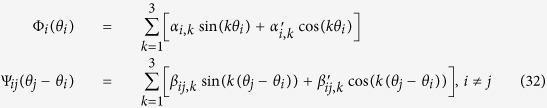


where all *α*_*i,k*_, 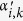, *β*_*ij,k*_, 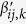, *k* = 1, 2, 3, uniformly distribute in the interval (−1, 1). Multiplicative noises Γ_*i*_(*t*) are simply chosen as


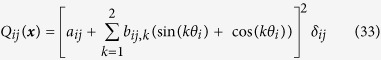


with *a*_*ij*_ and *b*_*ij,k*_ randomly and uniformly distributed in (0,1). In Fig. 5(a–c) the reconstruction results for different *m*_*i*_ are compared. According to the self-consistently checking condition, we conclude that *m*_*i*_ = 1, 2 are not suitable (see Fig. 5(a,b)) while the truncation at *m*_*i*_ = 3 is enough for correct network reconstruction (see the distribution of dots around the diagonal line in Fig. 5(c)). In Fig. 5(d) the inference results of *m*_*i*_ = 3 are compared with actual values of *A*_*ij*_. With all harmonic terms being taken into account, we achieve rather precise reconstruction with certain fluctuations. Moreover, by applying approaches of Eqs (13)(14) we can explore the statistical structures of noise and reveal multiplicative factors rather accurately (Fig. 5(e)).

In the above analysis we consider only dynamical and white noises, i.e., Γ(*t*) in Eq. (1), by assuming that all the node variables ***x**(t*) can be measured accurately. The analysis can be extended to measurement noises and colored noises, as we show in Supplementary Information. Moreover, due to noises and finite data length reconstruction errors are inevitable. Some facts influencing errors of inference are also briefly discussed in Supplementary Information. All discussion in this paper focused on reconstructions of homogeneous random networks. The HOCC method can be applied equally well to networks of different structures, including small-world and scale-free networks.

## Conclusions

In conclusion we have studied the problem of inferring noise-driven nonlinear dynamic networks with measurable data of node variables only. A high-order correlation computation (HOCC) method is proposed to unifiedly treat nonlinear dynamic structures, coupling topologies and statistics of additive and multiplicative noises in networks. This method treats network reconstruction by jointly considering three facts: choosing suitable basis and correlator vectors to expand nonlinear terms of networks; adjusting correlation time difference to decorrelate noise effects; and applying high-order correlations to derive linear matrix equations to infer nonlinear structures, topologies and noise correlation matrices. The HOCC algorithm has been theoretically derived, and its predictions are well confirmed by numerical results.

In biological, social and other crossing fields, we have huge amount of data available for analysis while often understand much less about the structures and dynamics producing these data. Nonlinear dynamics, mutual node interactions and noises often cooperate to yield various functions. Now with the development of the network reconstruction research, it is hopefully expected that we can explore hidden mechanisms, find underlying principles and reveal various unknown key parameters of practical dynamic networks by analyzing measurable output data only. These capabilities create a novel and significant perspective for understanding, modulating and controlling realistic network processes.

## Materials and Methods

### Derivation of Eqs (13) and (14)

Equations (13) and (14) can be derived as follows:


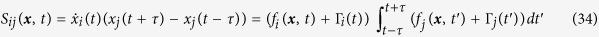


Its expectation on noise realizations reads


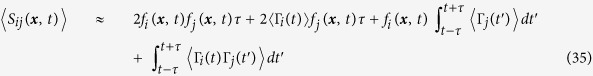


Since 〈Γ_*i*_(*t*)〉 = 〈Γ_*j*_(*t*)〉 = 0 and *τ* ≪ 1 we have


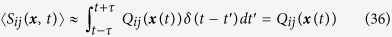


Considering the expansion of *Q*_*ij*_(***x***) on ***q***_*ij*_(***x***) bases


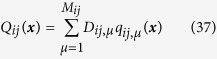


multiplying the two sides of Eq. (36) by basis 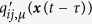 and making time average, we arrive at


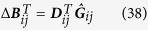


with Δ***B***_*ij*_ being vector having elements


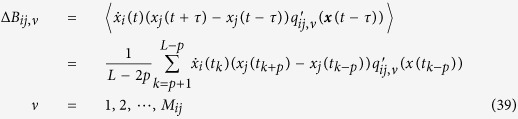


and 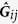 being matrix with elements


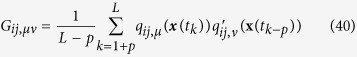


Finally we obtain


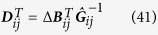


where all elements of vector Δ***B***_*ij*_ and matrix 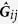 can be computed from available data and well defined bases *q*_*ij,μ*_, 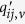 and vector ***D***_*ij*_ can thus be inferred by Eq. (41).

## Additional Information

**How to cite this article**: Yang, C. *et al*. Reconstruction of noise-driven nonlinear networks from node outputs by using high-order correlations. *Sci. Rep.* **7**, 44639; doi: 10.1038/srep44639 (2017).

**Publisher's note:** Springer Nature remains neutral with regard to jurisdictional claims in published maps and institutional affiliations.

## Supplementary Material

Supplementary Information
