# Supplementary material for: Reconstruction of noise-driven nonlinear networks from node outputs by using high-order correlations
Source: Sci Rep. 2017 Mar 21;7:44639. doi: 10.1038/srep44639 (PMC5359559; doi:10.1038/srep44639)
Supplement: Supplementary Information [file srep44639-s1.pdf]

Supplementary Information

Reconstruction of noise-driven nonlinear networks from node outputs by using high-order correlations

Chen Yang[1], **Zhang Zhaoyang**[2], **Chen Tianyu**[1], **Wang Shihong**[1,†], **and Hu Gang**[3,*]

[1]School of Sciences, Beijing University of Posts and Telecommunications - Beijing, China

[2]Faculty of Science, Ningbo University - Ningbo, China

[3]Department of Physics, Beijing Normal University - Beijing, China

[*]ganghu@bnu.edu.cn; [†] shwang@bupt.edu.cn

Effects of measurement noises and colored noises

Actually, practical measurements are often noisy, namely, the data measured are not exactly $x_i(t)$ in Eq.(1), but $y_i(t)$

$$y_i(t) = x_i(t) + \eta_i(t) \tag{S1}$$

with measured noises $\eta_i(t)$, $i = 1, 2, \cdots, N$, are also unknown. Inserting Eq.(S1) into (1), the network dynamics with both measurement and dynamical noises reads

$$\dot{y}_i(t) = f_i(\boldsymbol{x}) + \dot{\eta}_i(t) + \Gamma_i(t) \tag{S2}$$

where $\dot{\eta}_i(t)$ are defined as

$$\dot{\eta}_i(t_k) = \frac{\eta_i(t_{k+1}) - \eta_i(t_k)}{\Delta t} \tag{S3}$$

implied in the measured data $y_i(t_k)$, $k = 1, 2, \cdots, L$. In practice, noises have short while finite correlation times. Here we consider simplest additive and colored Gaussian noises in Eq.(S2) as

$$< \eta_i(t)\eta_j(t-\tau) > \;=\; \delta_{ij}\frac{P_{ij}}{\tau_m(i)}\exp(-|\tau|/\tau_m(i)) \tag{S4}$$

$$< \Gamma_i(t)\Gamma_j(t-\tau) > \;=\; \delta_{ij}\frac{Q_{ij}}{\tau_d(i)}\exp(-|\tau|/\tau_d(i)) \tag{S5}$$

For inferring field $\boldsymbol{f}(\boldsymbol{x})$ we exactly do the same as Eq.(4)-(11) by replacing $\boldsymbol{x}(t)$ by $\boldsymbol{y}(t)$ in all correlation computations. The time-distance τ is chosen such as $\tau > \tau_m, \tau_d$, so that the decorrelation of Eq.(9) is approximately valid for both dynamical and measurement noises, and we finally arrive at

$$\boldsymbol{A}_i^T = \boldsymbol{B}_i^T(-\tau)\hat{\boldsymbol{C}}_i^{-1} \tag{S6}$$

Unlike Eq.(11), here $\boldsymbol{B}_i$ and $\hat{\boldsymbol{C}}_i$ are computed with actually measured variables $\boldsymbol{y}(t)$, not the exact outputs of the network $\boldsymbol{x}(t)$. Here we show some numerical results of Eq.(S6) for the example of Lorentz system Eq.(16). In Fig.S1(a) we infer the Lorentz system Eq.(16) with measurement and dynamical noises both assumed to be white and additive, and do the same as Fig.2(c) by using Eq.(S6). The results justify the algorithm fairly well. However, with measurement noises errors in Fig.S1(a) look considerably larger than those in Fig.2 with dynamical noises only. The reason for these large errors can be easily understood. With high-frequency measurements ($\Delta t \ll 1$ in Eq.(3)), the measurement induced "dynamical noises" in

Eq.(S2) are amplified by a factor $1/\Delta t$ (see Eq.(S3)), and become much stronger than actual dynamical noises. However, this type of noises can be effectively reduced by a so-called smoothing method

$$\boldsymbol{y}_s(t_k) = \frac{1}{\Omega}\sum_{i=k}^{k+\Omega-1}\boldsymbol{y}(t_i) \tag{S7}$$

This smoothing method can dramatically reduce the intensity of measurement-induced driving $\boldsymbol{\eta}$. In Fig.S1(b) we smooth the data with $\Omega = 10$, the results of Eq.(S2) are greatly improved from those of Fig.S1(a).

The algorithm of Eq.(11) and Eq.(S6) can be also applied to colored noises. In Figs.S1(c) and (d) we present some results of reconstructions of a colored noise driven Lorenz system. The results of (c) is not good (large errors) while those of (d) are satisfactory, because τ used in (c) is smaller than the correlation time τ_d of noises while that in (d) is much larger than τ_d. Since τ_d is an unknown parameter, τ has to be selected by a self-consistent manner purely from the measurable data. In Fig.S1(e) we plot $|\Delta\boldsymbol{A}|$ vs τ where $|\Delta\boldsymbol{A}|$ is the $|A|$ difference between two adjacent tests with τ difference of $\Delta\tau = \tau(k+1) - \tau(k)$

$$|\Delta\boldsymbol{A}_i(k)| = \frac{1}{M}\sum_{j=1}^{M}|A_{ij}(k+1) - A_{ij}(k)| \tag{S8}$$

The τ actually used can be properly chosen when $|\Delta\boldsymbol{A}_i(k)|$ saturates to some small values. The τ chosen in (c) is too small that $|\Delta\boldsymbol{A}|$ decreases as τ sharply, while τ in (d) is proper, then $|\Delta\boldsymbol{A}|$ no longer changes much by further increasing τ.

Analysis of reconstruction errors

It is clear that for exact reconstruction finite truncations of M_i in Eq.(11) and M_{ij} in Eq.(13) must contain all expanded nonzero terms of field $f_i(\boldsymbol{x})$ and noise matrix element $Q_{ij}(\boldsymbol{x})$. If Eqs.(4) and (12) contain infinitely many nonzero terms, the expansions must converge and we must take infinite M_i and M_{ij} for obtaining exact solution. Under suitable truncations the HOCC algorithms Eqs.(11) and (13) are exact in the limits of truly white noise, infinitely large measurement frequency and infinitely large data sample.

$$\frac{1}{\Delta t}, \frac{1}{\tau} \to \infty;\ \frac{\Delta t}{\tau_d}, \frac{\Delta t}{\tau_m}, \frac{\tau}{\tau_m}, \frac{\tau}{\tau_d} \to \infty;\ L \to \infty \tag{S9}$$

In practice, all these quantities are finite, and the reconstruction method must have certain errors. If these quantities have finite while large values the method can approximately work with fairly high precision.

However, if any of above quantities is small, errors may be large and even the method fails. Similar arguments are also valid for truncations M_i and M_{ij}. In the following we briefly analyze how computation errors are influenced by some key facts.

Let us focus on network reconstruction of Eq.(26) with additive and white noises. An error quantity, average error, can be defined as

$$E = \sqrt{\frac{\sum_{i=1}^{N}\sum_{j=1}^{M_i}(A'_{ij}-A_{ij})^2}{\sum_i^N M_i}} \tag{S10}$$

(i) Effects of sample length L.

Sample size is obviously an important and practical quantity, closely related to computation errors. A rough and intuitive idea is: assuming computation errors to be random and mutually uncorrelated numbers, average error E should approximately decrease with sample length as $E \propto L^{-1/2}$. In Fig.S2(a) we compute errors by inferring model Eq.(26) with Fourier expansion (see red squares), and fairly well confirm this $E-L$ scaling.

(ii) Effects of network size N.

Intuitively, enlarging network size may make the reconstruction computation more complicated and then naturally yield larger errors. In Fig.S2(b) we investigate the effect of network size on reconstruction errors for model Eq.(26). To our surprise E is not sensitive to the variation of system size. Similar results are also observed for model Eq.(19) and other models. These observations can be understood as follows. For fixing other conditions including noise intensities, computation errors are roughly determined by average data length of per parameter to be determined. For large network size we have more unknown parameters while also data of more nodes. In Fig.S2(b) average data of per unknown parameter remains not considerably changed by changing N, and thus E remains similar values (with small fluctuations) for different N's.

(iii) Effects of choices of vector bases $Y_{i,\mu}(\boldsymbol{x})$.

Different choices of vector bases can dramatically change the reconstruction results. First, vector bases should be such chosen that the expansions of fields converge. Second, the convergence rate essentially determines the efficiency of reconstructions (higher efficiency means smaller computation errors and less computation consumption of reconstructions). It is still not very clear how to choose suitable vector bases for expansions by reading available data only. Various power expansions are generally used

for the first choice for data analyses without knowing any particular properties of systems, or Fourier expansions can be used if outputs under the investigation have phase angle nature.

In Fig.S2(c) we use power expansions to study Kuramoto model Eq.(26). The HOCC method still works. With the same data as Fig.5, the network structure can be satisfactorily inferred (Fig.S2(c)), however, with larger fluctuations compared to the case of Fourier expansions (subgraph in Fig.S2(c)). In Fig.S2(a) we do the same as red squares with power expansion applied (blue disks), and the results of both basis sets are compared. The scaling rule $E \propto L^{-1/2}$ is approximately kept for applications of both sets. For the same observation time average errors for power expansion are much larger than those of the Fourier expansion, (e.g., at the $L = 10^7$, the former E is more than 6 times larger than that of the latter). The reason is that for the Kuramoto model power expansion converges much slower than Fourier expansion, and the reconstruction computation needs to take more unknown parameters into account.

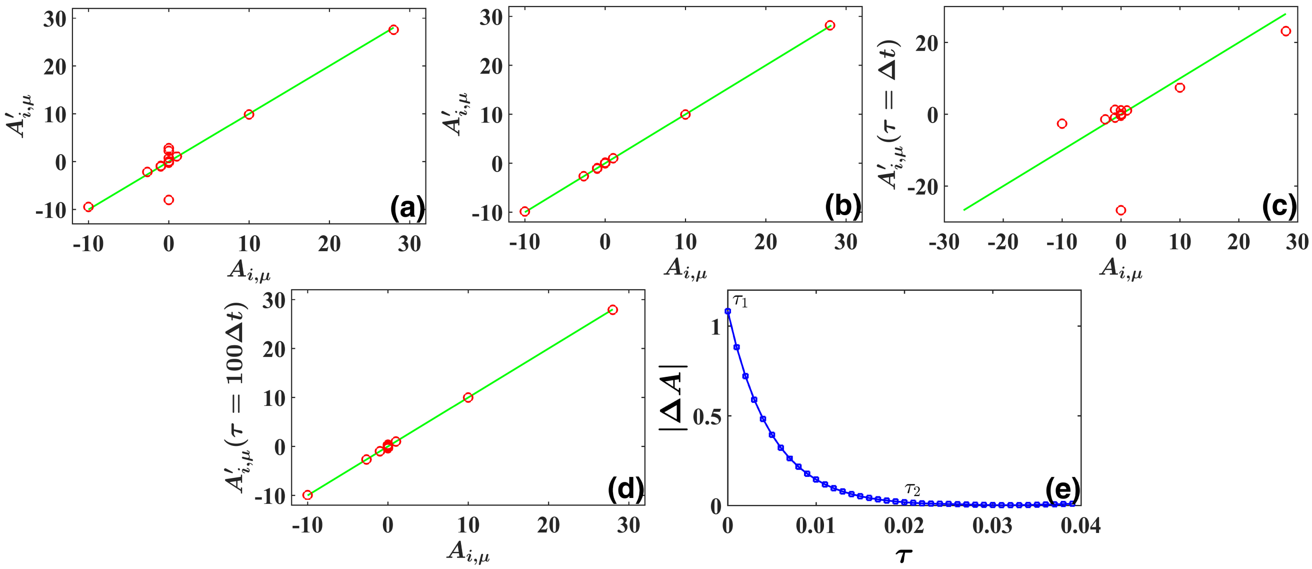

Figure S1. Applications of the HOCC method to reconstructions of Eq.(16) with measurement noises and colored noises. (**a**) Reconstruction results obtained by Eq.(S6) with the same time sequences as in Fig.1(a) contaminated by additive white measurement noises of intensity $P = 3.0$ in Eq(S4). The results are satisfactory, but bear considerably larger errors than those in Fig.2(c). (**b**) The same as (a) after data smoothing of Eq.(S7) with a window of $\Omega = 10$. The reconstruction precision has been dramatically improved. (**c**)(**d**) Inference of Lorenz model driven by Gaussian colored noises by computing Eq.(11) with $\tau_1 = \Delta t$ ((c)) and $\tau_2 = 100\Delta t$ ((d)). The results in (c) are poor while they are very good in (d). $\tau_d = 0.005 = 25\Delta t$ and dynamic noises $Q = 200$. With properly chosen τ, $\tau > \tau_d$, the algorithm Eq.(11) works well for colored noises. (**e**) Difference $|\Delta A|$ in Eq.(S8) plotted vs. τ. The suitable τ can be chosen such at which $|\Delta A(k)|$ saturates to small values.

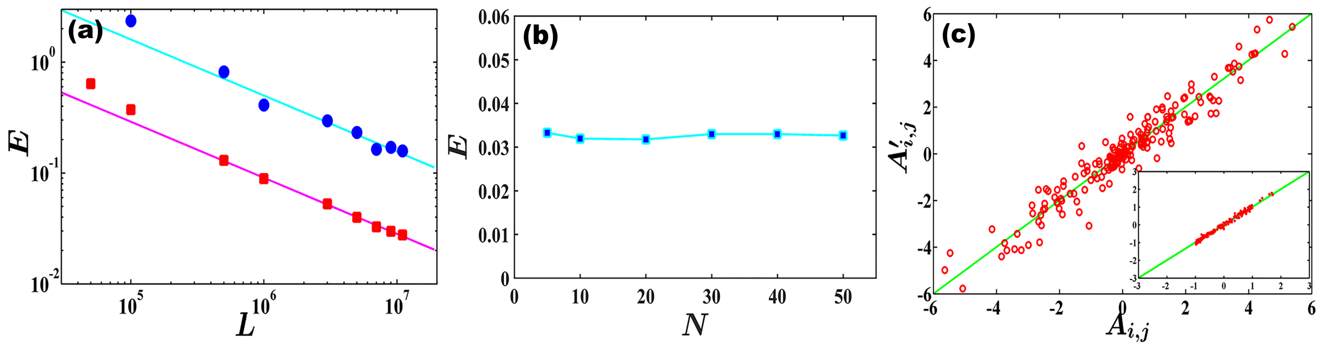

Figure S2. Influences of different factors on reconstruction errors, including data length L ((a)), system size N ((b)) and selected vector bases $Y_{i,\mu}(\boldsymbol{x})$ ((a), (c)). Kuramoto Model of Eq.(26) driven by additive white noises is adopted with $\Delta t = 5 \times 10^{-4}$ and $Q_{ij} \in \delta_{ij}(0.5, 1.5)$. Average error E is defined in Eq.(S10). (**a**) E plotted vs. L for reconstructions by using Fourier expansion set (red squares) and power expansion set (blue disks). The two straight lines have slope of $-1/2$, and most of $E-L$ plots are around the lines. $N = 5$. Φ_i and Ψ_i are truncated at $m_i = 3$ for Fourier expansion while $m_i = 13$ for Taylor expansion. For Taylor expansion all θ_i and $(\theta_j - \theta_i)$ are modulo 2π, $x_i = [\theta]_{2\pi}$, $(x_j - x_i) = [\theta_j - \theta_i]_{2\pi}$. (**b**) E plotted vs. N. $L = 2 \times 10^7$. It is obvious that computation errors are insensitive to network size. (**c**) Reconstruction results of Eq.(26)by using with power expansion. $N = 5$ and $L = 10^6$. The network has been reconstructed approximately with, however, much larger errors than those by using Fourier expansion set (plots in the subgraph).
